# Reproductive health status of adolescent mothers in an Iranian setting: a cross-sectional study

**DOI:** 10.1186/s12978-022-01396-9

**Published:** 2022-04-02

**Authors:** Maryam Zare, Afrouz Mardi, Mozhgan Gaffari-moggadam, Nazila Nezhad-dadgar, Malek Abazari, Atefeh Shadman, Arash Ziapour

**Affiliations:** 1 Department of Nutrition, Khalkhal University of Medical Sciences, Khalkhal, Iran; 2 Department of Public Health, School of Health, Ardabil University of Medical Science, Ardabil, Iran; 3 Department of Health Care Services and Health Education, School of Health, Health Education and Health Promotion, Ardabil University of Medical Science, Ardabil, Iran; 4 School of Health, Ardabil University of Medical Science, Ardabil, Iran; 5 School of Medicine, Ardabil University of Medical Science, Ardabil, Iran; 6 Social Development and Health Promotion Research Center, Health Institute, Kermanshah University of Medical Sciences, Kermanshah, Iran

**Keywords:** Adolescents, Mothers, Reproductive health

## Abstract

**Background:**

In low and middle-income countries (LMICs), where millions of women give birth before the age of 18, the reproductive health status of married adolescent mothers, including family planning, sexual, psychosocial, and maternal health, remains a significant and recurring phenomenon. As a result, the purpose of this study was to assess the reproductive health status of married adolescent mothers who sought treatment at Ardabil health care centers in 2019.

**Methods:**

A cross-sectional study was carried out in five health centers in Ardabil, Iran. This research included 312 married adolescent mothers who were under 19 years old. Health workers who asked questions of each participant completed a demographic questionnaire and the Reproductive Health Assessment Scale for Married Adolescent Women, and all data were self-reported. Univariate and multivariate linear regressions were used to determine risk factors associated with reproductive health scores. The data was examined using statistical software (SPSS version 20).

**Results:**

The mean age (years) of the respondents was 16.41 ± 0.85, the mean age of their husbands was 24.18 ± 2.29, and the mean age of their marriage was 15.06 ± 1.15. In this study, adolescent mothers had an average reproductive health score of 63.78 ± 11.06. There was a significant association between reproductive health status and age, education, husband’s age and education, and contraceptive methods among married adolescent mothers (p < 0.05).

**Conclusion:**

The research findings indicated that adolescent mothers had an average level of reproductive health. Several socio-demographic characteristics, including age, education, gravida, and contraception, were linked to reproductive health scores. Programmers and policymakers should prioritize improving the reproductive health of adolescent mothers through education and increasing women’s and spouses’ knowledge and awareness.

## Plain language summary

In LMICs, the health status of adolescent mothers remains unstudied in a systematic way. This study aims to evaluate the reproductive health of married adolescent mothers. A cross-sectional study of adolescent mothers was conducted at five Ardabil health centers. A total of 312 adolescent mothers under the age of 19 were selected to complete a survey regarding their health and contraceptive awareness. The mothers’ reproductive health status was rated as average. Age, education, husband’s age and education, gravida, and contraceptive methods significantly impacted reproductive health. Increased education among women and their spouses, as well as pregnancy prevention, appear to be essential for safeguarding the health of adolescent mothers. This study recommends conducting qualitative research on reproductive health and contraception in the cultural context.

## Introduction

Adolescence is described as the age range of 10 to 19 years. Adolescents are classified into two groups: early adolescent (ages 10–14 years) and late adolescent (ages 15–19 years). Adolescents constitute approximately 20% of the global population, with the majority living in LMICs [1, 2]. Globally, one in every six adolescent girls between the ages of 15 and 19 is married, and over 700 million girls marry as adolescents, with more than a third being under the age of 15. Adolescent girls’ pregnancy, as well as their reproductive health, is a concern in many LMICs. Over 12 million girls in LMICs give birth before the age of 18 [3]. Typically, these young women are pressured to have children soon after marriage for socio-cultural reasons such as proving their identity and fertility as adult females, improving their status in the spouse’s family, avoiding loneliness, and having limited decision-making power [4, 5].

Early marriage has a negative impact on health and nutrition, individual and community well-being, sexual and reproductive health, and maternal morbidity and mortality [6, 7]. In several LMICs, maternal causes are still among the top causes of mortality among adolescents aged 10–24 years [8]. Over 16 million births occur each year among women aged 15 to 19, accounting for 11% of all deliveries [9]. In 2019, 29.8 million of the 300 million women aged 15 to 19 did not use contraception, and 15.0 million had unmet family planning requirements [10].

Although early marriage is less common in Iran than in many other LMICs, it is still prevalent; in 2018, it was 3% for girls under the age of 15 and 17% for those under the age of 18 [11, 12]. Even though the legal marriage age in Iran is 13 years for girls, in some areas, such as Ardabil province, the average rate of marriage before the age of 15 has been reported to be as high as 9% [13, 14]. Despite the fact that most adolescent women were reluctant to become pregnant, they did not use contraception because they were unaware of the contraceptive methods, had misconceptions about them or were pressured to conceive by family members [4].

Promoting women’s reproductive health is one of the 2030 Sustainable Development Goals [10, 15]. Meanwhile, the critical and ongoing problems in LMICs are the lack of information about adolescent girl’s reproductive health status, as well as their apparent lack of access to Sexual and Reproductive Health services (SRH), and the general societal disregard for their health, including; family planning, sexual, psychosocial, and maternal health [16, 17]. Research and information are scarce on reproductive health among adolescent mothers [17]. The purpose of this study was to determine the reproductive health status of married adolescent mothers attending Ardabil health care centers in 2019 in order to gain a better understanding of their reproductive health status and contraception.

## Methods

### Study design and settings

This analytic cross-sectional study was conducted in Ardabil province between January and June 2019 on married adolescent mothers who visited health care clinics affiliated with Ardabil University of Medical Sciences. Ardabil province is located in northwestern Iran, which has an estimated population of 1,300,000 people who speak Azeri [14] and is divided into five regions. Ardabil, the capital of Ardabil province, was selected for this study.

### Study population and sample size

The stratified cluster sampling was used to choose health care centers from five city regions (north, south, center, east, and west), and a health care center was randomly selected from each region. The convenience sample approach was used to recruit mothers in the study.

First, the sample size (n = 306) was determined using the following formula; (p = 15%), according to pilot before study, significance level 5% (α = 0.05), (d = 0.04).

$$n = \frac{{Z_{{1 - \frac{\alpha }{2}}}^{2} p(1 - p)}}{{d^{2} }}$$

Finally, 312 married adolescent mothers were included in the study.

The inclusion criteria included being a married woman under the age of 19 who had given birth at least once. Exclusion criteria were; addiction and suffering from any chronic disease, which was self-declared.

### Data collection and measurements

The respondents were informed of the study’s purpose by the staff of the healthcare centers. Staff read the questionnaires to participants and filled out a socio-demographic questionnaire and the Reproductive Health Assessment Scale for Married Adolescent Women (RHAS-MAW) for them. Age, education, employment, age at marriage, gravida, parity, type of delivery, number of children, and spouse’s demographic information were included in the socio-demographic questionnaire. All information was self-reported by the participants. The RHAS-MAW questionnaire gathered information about the sexual domain, pregnancy and delivery, psychosocial domain, and family planning.

The contraceptive methods were categorized into two groups based on data from the RHAS-MAW questionnaire: modern methods (oral contraceptive pill (OCP), IUD, condom, depot-medroxyprogesterone acetate (DMPA)), and traditional methods (withdrawal, rhythm, calendar method).

Mardi et al. [18] developed the RHAS-MAW questionnaire in 2018. This scale was established with 27 items and four domains as the outcome of an exploratory mixed-methods study: Questions 1–7 dealt with the sexual dimension (for example, “My first sexual experience was unpleasant”), questions 8–14 dealt with pregnancy and childbirth (for example, “I think pregnancy completes me”), questions 15–22 were concerned with the psychosocial dimension (for example, “I have no decision-making power in my married life”), and questions 23–27 were concerned with family planning (for instance: “I know about emergency contraception”). A 5-point Likert scale was employed for these questions. The answers included: strongly disagree, disagree, indifferent, agree, and strongly agree, with each possible answer to a question receiving a score between 0 and 4.

Consequently, the RHAS-MAW questionnaire comprised a raw score of between 0 and 108 points. This raw score was then converted to a range of 0 to 100. This scoring range is classified as poor (0–50), fair or average (50.1–66), good (66.1–84), and excellent (84.1–100). Cronbach’s alpha (0.90) for the entire scale and test–retest reliability was established using a two-week interval Intraclass Correlation Coefficient (ICC = 0.996, p < 0.001). This questionnaire was validated based on content, face, and concept validity.

### Data analysis

The frequency and percentage of categorical variables and the mean and standard deviation (SD) of all continuous variables were calculated. The independent samples t-test and ANOVA were used to compare reproductive health scores by demographic characteristics. Univariate and multivariate linear regressions were used to determine risk factors associated with reproductive health scores. The multivariate linear regression included all significant factors from the univariate linear regression. The Kolmogorov–Smirnov test was used to determine normalcy in this study. The criterion for significance was two-sided and 0.05.

SPSS Version 21 software (SPSS Inc., IL, Chicago, USA) was used for statistical analysis.

### Ethical considerations

Everyone who took part was guaranteed that their information would be kept secret and anonymous. Participation in the study was voluntary, and prior to the study, all respondents provided written consent. The Ardabil University of Medical Sciences' Ethical Committee accepted the study (IR.ARUMS.REC.1397.2.3).

## Results

### Socio-demographic characteristics

Table 1 displays the respondents’ socio-demographic characteristics and their reproductive health scores. The respondents’ mean age was 16.47 ± 0.85 years, with a range of 13 to 18 years, and more than half (61.2%) were 17 years old. The mean age of the respondents upon marriage was 15.06 ± 1.15, ranging from 12 to 17 years, while their husbands’ mean age was 24.18 ± 2.29, ranging from 19 to 35 years. The majority of respondents (42%) had completed junior high school.

**Table 1:** Association between socio-demographic characteristics and mean reproductive health scores

Variable	Socio-demographic characteristics	Reproductive health score	P-value
	N (%)	Mean (SD)	
Age			
≤ 15	49 (15.7)	66.40 (10.39)	0.07
< 15	263 (84.3)	63.29 (11.13)	
Marriage age			
≥ 13	21 (6.7)	64.00 (9.43)	0.409
14–15	176 (56.4)	64.47 (11.43)	
> 15	115 (36.9)	62.67 (10.75)	
Education			
Elementary school	49 (15.7)	61.89 (9.74)	0.005
Junior high school	131 (42)	61.86 (12.02)	
Senior high school	89 (28.5)	66.61 (10.27)	
High school diploma	43 (13.58)	65.90 (9.60)	
Gravida			
1	223 (71.5)	65.75 (10.37)	0.003
2	83 (26.6)	61.64 (10.90)	
3	6 (1.9)	61.84 (12.69)	
Contraceptive method			
Modern	85 (30.36)	61.34 (10.1)	0.012
Traditional	110 (39.28)	59.06 (10.38)	
Nothing	85 (30.36)	59.58 (10.68)	
Para			
1	272 (87.2)	65.01 (11.84)	0.007
2	40 (12.8)	59.62 (16.06)	
Age of spouse			
>20	68 (21.8)	59.35 (12.61)	< 0.001
20–25	153 (49)	63.92 (9.87)	
> 25	91 (29)	66.84 (10.75)	
Education of spouse			
Elementary school	79 (25.3)	58.30 (10.25)	< 0.001
Junior high school	79 (25.3)	65.12 (11.89)	
Senior high school	41 (13.1)	64.75 (10.19)	
High school diploma	84 (26.9)	67.15 (9.74)	
College	29 (9.3)	63.89 (10.83)	
Type of delivery			
Vaginal	65 (40.12)	65.01 (11.84)	0.158
Cesarean	97 (59.87)	59.62 (16.06)	

### Reproductive health

The majority of respondents (87.5%) had a single delivery, while the remainder had two, with 54.8% concluding through cesarean section. Only 30.36% of married adolescent mothers used modern contraceptive methods. Traditional methods were utilized by 39.28%, whereas no contraception was used by 30.36%. With a range of 31 to 78, the mean reproductive health score for married adolescent mothers was 63.78 ± 11.06. This research showed a statistically significant relationship between education, contraception, gravida, para, spouse age, and spouse education and reproductive health scores (p < 0.05; Table 1).

There was no statistically significant relation between age, marriage age, husband’s occupation, type of delivery, living location, and reproductive health status among married adolescent mothers (p > 0.05; Table 1).

The mean score for each domain of the RHAS-MAW questionnaire is shown in Table 2. The mean overall score for individuals on this questionnaire was 63.78 (SD = 11.06), and this score was considered an average reproductive health status.

**Table 2:** The mean score of the domain specific aspects of the reproductive health score of the respondents

Reproductive health score	Items	Minimum	Maximum	Mean (SD)
Sexual	7	6	28	18.21 (5.10)
Pregnancy and childbirth	7	6	25	16.55 (4.27)
Psycho-social	8	4	29	17.11 (4.51)
Family planning	5	3	18	11.90 (3.66)
Total	27	31	87	63.78 (11.06)

Univariate linear regression was employed to compare reproductive health scores across factors. There were statistically significant variations in reproductive health scores based on age, level of education, gravida, contraceptive methods, spouse’s age, and degree of education (p < 0.05; Table 3). Multiple linear regression indicated four variables that were connected with reproductive health score: age, level of education, age, and level of education of spouse (p < 0.05; Table 3). Women’s age increasing from 15 to 18 years was associated with a decrease in reproductive health scores (beta = − 2.18), whereas their spouse’s age increase was associated with an increase in reproductive health scores (beta = 0.952). Women with a senior high school education had a higher reproductive health score than those with an elementary or high school education (beta = 4.60). Married adolescent mothers who had two pregnancies exhibited a poorer reproductive health score (beta = − 4.47) than women who had one pregnancy. Women with educated husbands and women who did not use contraception scored higher on reproductive health (beta = 5.04 and 5.93, respectively; Table 3).

**Table 3:** Simple and multiple linear regression of the association between demographic characteristics and reproductive health scores

Variable	Univariate		Multivariate	
	Beta (95%CI)	P-value	Beta (95%CI)	P-value
Age	− 1.55 (− 2.98 to − 012)	0.033	− 2.18 (− 2.8 to—0.211)	0.006
Age at marriage	− 0.988 (− 1.81 to 0.05)	0.068		
Spouse age	0.876 (0.52 to 1.22)	< 0.001	0.952 (0.60 to 1.30)	< 0.001
Education				
Elementary school	Ref			
Junior high school	− 0.035 (− 1.26 to − 0.08)	0.985	− 0.732 (− 5 to 2.21)	0.679
Senior high school	4.001 (1.29 to 6.70)	0.004	4.60 (1.99 to 7.21)	0.001
High school diploma	2.36 (− 1.24 to 5.98)	0.073	3.713 (− 2.55 to 6.43)	0.11
Gravida				
1	Ref			
2	− 4.47 (− 6.89 to − 2.05)	< 0.001	− 3.23 (− 5.77 to − 0.68)	0.01
3	4.43 (− 007 to 8.87)	0.057	0.975 (− 3.50 to5.63)	0.691
Contraceptive				
Modern	2.78 (− 1.5 to 3.96)	0.65		
Traditional	3.57 (081 to 6.32)	0.01	2.26 (− 2.06 to 3.47)	0.16
Nothing	5.57 (1.2 to 6.35)	0.001	3.29 (− 0.18 to 6.19)	0.065
1	Ref			
2	− 2.11 (− 4.37 to 0.14)	0.066		
Elementary school	Ref			
Junior high school	6.82 (1.85 to 7.56)	< 0.001	6.14 (2.65 to 6.89)	< 0.001
Senior high school	6.45 (1.65 to 7.32)	0.002	4.62 (1.8 to 5.93)	0.031
High school diploma	8.85 (2.1 to 9.2)	< 0.001	5.04 (2.10 to 6.20)	0.004
College	5.93 (1.52 to 6.54)	0.016	2.52 (− 0.79 to 2.57)	0.269
Vaginal	Ref			
Cesarean	− 2.47 (− 5.91 to 0.96)	0.158		

## Discussion

According to the findings of this study, the married adolescent mothers had an average level of reproductive health.

Our data also revealed a relationship between the mother’s age and educational attainment and the spouse’s age and educational attainment, gravida, contraceptive methods, and reproductive health status. As the mother’s age increased from 15 to 18, the reproductive health score decreased but increased as the husband’s age increased. This is most likely due to the increased risk of adverse effects associated with pregnancy, childbirth, and contraception as women’s ages increase among women who are still adolescent. If the individual had been over 18, the outcome might have been different.

Ganchimeg et al. observed that adolescent women (13–19 years old) had more complications with pregnancy and delivery than young women (20–24 years old) [19]. According to the findings of this study, men’s aging is often accompanied by increased knowledge, education, and maturity, which has a positive impact on women’s reproductive health. Most of the women in this study had completed junior high school, whereas their spouses had completed high school. The educational level of mothers and their partners influences their reproductive health.

Mothers’ mean reproductive health ratings improved as women’s and husbands’ educational levels increased. Bandari et al. report that educational attainment is one of the practical determinants that impact adolescent women’s health [20]. Meanwhile, according to the World Health Organization (WHO) research, increasing educational possibilities for girls boosts their health and reduces the likelihood of early marriage and its consequences [21].

Another research finding was that approximately one-third of non-pregnant adolescent mothers used modern contraceptive methods, while the remainder used traditional methods or did not use contraception at all. Despite their desire to avoid pregnancy, over 200 million women, primarily in low- and middle-income countries (LMICs), do not use any effective contraception [22]. Contraceptive uptake and usage patterns among adolescent and adult women, on the other hand, differ significantly. Adolescent women are less likely to use contraceptives than adults. This discrepancy may be attributed to adolescents’ lack of information and experience, notably their lack of decision-making freedom [23].

Furthermore, most adolescent women desire to have children, mainly male offspring, to strengthen their place and identity within their spouse’s family [6]. A lack of educational, social, and cultural barriers to accessing family planning services limits contraceptive use among young women shortly after marriage [24, 25].

This study’s findings also revealed a relation between contraceptive use and reproductive health status. As a result, women who did not use contraception had better reproductive health than those who did. Despite this, multiple studies have demonstrated that taking contraception decreases maternal mortality by up to 44% and improves women’s health by reducing unexpected pregnancies and unsafe abortions [26]. This conclusion could be related to specific contraceptive concerns for adolescent mothers who are unaware of how to use the methods and care for themselves or poor physical health or dissatisfaction with their current role among those using contraception to avoid another birth. Due to misconceptions, some adolescent women attribute various issues or disorders to contraception (4).

The majority of adolescent mothers in this study had only one pregnancy and delivery, and gravida and parity were significantly associated with their reproductive health. Consequently, mothers with one child exhibited better reproductive health status than those with two children. Many studies revealed that pregnancy and delivery during adolescence modify health status and impact their physical, mental, and social health [27, 28].

As previously noted, several of the adolescent women in our study had two children. According to Aguilar et al., most of these cases have their second pregnancy within two years and experience the adverse side effects of these recurring pregnancies and deliveries until the end of their lives [29].

Meanwhile, more than half of the women gave birth by cesarean section, which might be related to prenatal complications [30] or pelvic disproportion [31].

In some studies, normal vaginal deliveries rather than cesarean sections were documented among adolescents, which might be due to a lack of or limited access to a gynecological surgeon or facility for cesarean section [27].

In this research, there was no statistically significant relationship between the type of delivery and the reproductive health status of adolescent mothers. In any event, it can be explained by the fact that adolescent childbearing has consequences in any event.

## Strength and limitations

The findings of this study identified several variables associated with the reproductive health score encountered by adolescent mothers in Ardabil City. Recognizing these challenges enables activities to improve the health of these vulnerable women.

However, there were certain drawbacks to this study. Several individuals did not intend to respond to some questions, but their trust in the staff mitigated this limitation.

## Conclusion

The study aimed to examine the reproductive health of adolescent mothers in Iranian society. The findings indicated that married adolescent mothers’ reproductive health was average. They must understand their rights and privileges regarding reproductive and sexual health. Our findings underscored the importance of promoting reproductive health among them by increasing their and their spouses’ knowledge and awareness of contraceptive methods, as well as their ability to use them. Preventing adolescent marriage and childbearing should be a top priority for healthcare providers and policymakers.

## Data Availability

Data for this study was sourced from this project: IR.ARUMS.REC.1397.2.3.

## Abbreviations

ANOVA: Analysis of variance
DMPA: Depot-medroxyprogesterone acetate
ICC: Intraclass Correlation Coefficient
LMICs: Low and middle-income countries
OCP: Oral contraceptive pill
RHAS-MAW: Reproductive Health Assessment Scale for Married Adolescent Women
SD: Standard deviation.
WHO: World Health Organization

## References

[1] United Nations DoEaSA, Population Division Monitoring global population trends. 2017. http://www.un.org/en/development/desa/population/publications/index.shtml.

[2] Akuiyibo S, Anyanti J, Idogho O, Piot S, Amoo B, Nwankwo N (2021). Impact of peer education on sexual health knowledge among adolescents and young persons in two North Western states of Nigeria. Reprod Health.

[3] Unicef. Investing in a safe, healthy and productive transition from childhood to adulthood is critical 2018. https://data.unicef.org/topic/adolescents/overview/.

[4] Mardi A, Ebadi A, Shahbazi S, Moghadam ZB (2018). Factors influencing the use of contraceptives through the lens of teenage women: a qualitative study in Iran. BMC Public Health.

[5] Ketema H, Erulkar A (2018). Married adolescents and family planning in rural ethiopia: understanding barriers and opportunities. Afr J Reprod Health.

[6] Wodon Q, Male C, Nayihouba A, Onagoruwa A, Savadogo A, Yedan A, et al. Economic impacts of child marriage: global synthesis report. 2017.

[7] Mardi A, Ebadi A, Moghadam ZB, Shahbazi S (2018). Perceptions of teenage women about marriage in adolescence in an Iranian setting: A qualitative study. Electron Phys.

[8] Ward JL, Azzopardi PS, Francis KL, Santelli JS, Skirbekk V, Sawyer SM (2021). Global, regional, and national mortality among young people aged 10–24 years, 1950–2019: a systematic analysis for the Global Burden of Disease Study 2019. Lancet.

[9] Sawyer SM, Afifi RA, Bearinger LH, Blakemore S-J, Dick B, Ezeh AC (2012). Adolescence: a foundation for future health. Lancet.

[10] Kantorová V, Wheldon MC, Dasgupta AN, Ueffing P, Castanheira HC (2021). Contraceptive use and needs among adolescent women aged 15–19: Regional and global estimates and projections from 1990 to 2030 from a Bayesian hierarchical modelling study. PLoS One..

[11] Kohan S, Allahverdizadeh S, Farajzadegan Z, Ghojazadeh M, Boroumandfar Z (2021). Transition into the sexual and reproductive role: a qualitative exploration of Iranian married adolescent girls’ needs and experiences. Reprod Health.

[12] UNICEF. Child marriage is a violation of human rights, but is all too common 2018. https://data.unicef.org/topic/child-protection/child-marriage/.

[13] Statistics of vital events, https://www.sabteahval.ir/Upload/Modules/Contents/asset99/e-g-94.pdf. IAf. Accessed 10 May 2017.

[14] Atabaki T. Azerbaijan: ethnicity and autonomy in twentieth-century Iran. British Academy Press; 1993.

[15] https://sustainabledevelopment.un.org/topics DoEaSASDTAf.

[16] Coast E, Jones N, Francoise UM, Yadete W, Isimbi R, Gezahegne K (2019). Adolescent sexual and reproductive health in Ethiopia and Rwanda: a qualitative exploration of the role of social norms. SAGE Open.

[17] Yount KM, Krause KH, Miedema SS (2017). Preventing gender-based violence victimization in adolescent girls in lower-income countries: Systematic review of reviews. Soc Sci Med.

[18] Mardi A, Ebadi A, Behboodi-Moghadam Z, Abazari M, Nezhad-Dadgar N, Shadman A (2021). Developing and psychometric evaluation of a reproductive health assessment scale for married adolescent women: an exploratory mixed-method study. Iran J Nurs Midwifery Res.

[19] Ganchimeg T, Ota E, Morisaki N, Laopaiboon M, Lumbiganon P, Zhang J (2014). Pregnancy and childbirth outcomes among adolescent mothers: a W orld H ealth O rganization multicountry study. BJOG..

[20] Bhandari SD, Joshi S (2016). Perception and perceived experiences about prevention and consequences of teenage pregnancy and childbirth among teenage mothers: a qualitative study. J Adv Acad Res.

[21] Chandra-Mouli V, Camacho AV, Michaud P-A (2013). WHO guidelines on preventing early pregnancy and poor reproductive outcomes among adolescents in developing countries. J Adolesc Health.

[22] Bongaarts J, Cleland J, Townsend JW, Bertrand JT, Gupta MD (2012). Family planning programs for the 21st century.

[23] Blanc AK, Tsui AO, Croft TN, Trevitt JL (2009). Patterns and trends in adolescents' contraceptive use and discontinuation in developing countries and comparisons with adult women. Int Persp Sex Reprod Health..

[24] Adams MK, Salazar E, Lundgren R (2013). Tell them you are planning for the future: gender norms and family planning among adolescents in northern Uganda. Int J Gynecol Obstet.

[25] Santos KA (2012). Teenage pregnancy contextualized: understanding reproductive intentions in a Brazilian shantytown. Cad Saude Publica.

[26] Behboodi-Moghadam Z, Salsali M, Eftekhar-Ardabily H, Vaismoradi M, Ramezanzadeh F (2013). Experiences of infertility through the lens of Iranian infertile women: a qualitative study. Japan J Nurs Sci..

[27] NeJhaddadgar N, Ziapour A, Abbas J, Mardi A, Zare M (2020). Correlation between general health and sexual function in older women in an Iranian setting. J Educ Health Prom..

[28] Nove A, Matthews Z, Neal S, Camacho AV (2014). Maternal mortality in adolescents compared with women of other ages: evidence from 144 countries. Lancet Glob Health.

[29] Aguilar Rivera AM, Cortez R. Family planning: the hidden need of married adolescents in Nepal. 2015.

[30] Kalhor M, Aj N, Alipour M, Eghdam PF (2015). Comparison of pregnancy and delivery outcomes in teenage mothers and primiparas referring to Kowsar Teaching Hospital in Qazvin in 2012–2013. Razi J Med Sci.

[31] Malabarey OT, Balayla J, Abenhaim HA (2012). The effect of pelvic size on cesarean delivery rates: using adolescent maternal age as an unbiased proxy for pelvic size. J Pediatr Adolesc Gynecol.

